# Waist-to-Height Ratio as a Simple Anthropometric Marker for Identifying Individuals at High Risk of MASLD: A Large Population-Based Analysis Using the Fatty Liver Index

**DOI:** 10.3390/metabo16040246

**Published:** 2026-04-04

**Authors:** Ángel Arturo López-González, Pedro Juan Tárraga López, Mónica Silu Piña Dabreu, Lluis Rodas Cañellas, Carla Busquets-Cortés, José Ignacio Ramírez-Manent

**Affiliations:** 1 Faculty of Dentistry, ADEMA-University School, University of the Balearic Islands, 07009 Palma, Spain; 2 Faculty of Medicine, University of Castilla La Mancha (UCLM), 02008 Albacete, Spain; 3 Balearic Islands Health Service, 07010 Palma, Spain

**Keywords:** fatty liver, anthropometry, waist circumference, body mass index, metabolic syndrome, risk assessment

## Abstract

Background: Metabolic dysfunction-associated steatotic liver disease (MASLD) is the most common chronic liver disease worldwide and represents a major component of the global burden of metabolic disorders. Simple anthropometric markers capable of identifying individuals at increased risk of hepatic steatosis are of considerable interest for population-level screening. Methods: In this cross-sectional population-based study, we evaluated the performance of waist-to-height ratio (WtHR) for identifying individuals with a high Fatty Liver Index (FLI ≥ 60), a widely used surrogate marker of hepatic steatosis. The study included 146,318 adult participants with available anthropometric and biochemical data. Discriminatory performance was assessed using receiver operating characteristic (ROC) curve analysis. Optimal WtHR thresholds were determined using the Youden index. Associations between WtHR and high FLI were evaluated using age-adjusted logistic regression models. Non-linear relationships were explored using restricted cubic spline models. Additional analyses included a comparison with body mass index (BMI) and waist circumference, decision curve analysis, and subgroup analyses across age and BMI strata. Results: The prevalence of high FLI in the study population was 18.1%. WtHR demonstrated excellent discriminatory performance, with area under the ROC curve (AUC) values of 0.908 (95% CI 0.906–0.910) in men and 0.972 (95% CI 0.971–0.974) in women. Optimal WtHR thresholds for identifying individuals with high FLI were 0.52 in men and 0.53 in women. Each 0.01 increase in WtHR was strongly associated with higher odds of high FLI (OR 1.56 in men and 1.69 in women). Restricted cubic spline analysis demonstrated a non-linear relationship, with a marked increase in predicted probability of high FLI above WtHR values of approximately 0.50–0.52. WtHR showed discriminatory performance comparable to BMI and waist circumference and maintained strong associations with high FLI across age groups and BMI categories. Conclusions: Waist-to-height ratio is a simple anthropometric marker strongly associated with a high Fatty Liver Index in a large population-based cohort. Given its simplicity, low cost, and ease of calculation, WtHR may represent a practical screening indicator for identifying individuals at increased risk of MASLD-related phenotypes in both clinical practice and population health strategies.

## 1. Introduction

Metabolic dysfunction-associated steatotic liver disease (MASLD), previously known as non-alcoholic fatty liver disease (NAFLD), has become the most prevalent chronic liver condition worldwide and represents a major component of the global burden of metabolic disease. Recent epidemiological estimates suggest that approximately 25–30% of the adult population globally has hepatic steatosis, with substantially higher prevalence among individuals with obesity, type 2 diabetes, and metabolic syndrome [1,2,3,4]. Beyond its hepatic manifestations, MASLD is increasingly recognized as a multisystem disease associated with increased risks of cardiovascular disease, chronic kidney disease, extrahepatic malignancies, and premature mortality [5,6,7,8].

In 2023, an international consensus proposed the adoption of the term MASLD to better reflect the central role of metabolic dysfunction in the pathogenesis of hepatic steatosis [9]. This change underscores the close relationship between liver fat accumulation and systemic metabolic abnormalities, including visceral adiposity, insulin resistance, and chronic low-grade inflammation. From a clinical and public health perspective, this conceptual shift highlights the importance of identifying individuals at increased metabolic risk before the development of advanced liver disease [10].

Despite the growing clinical relevance of MASLD, population-level identification of individuals with hepatic steatosis remains challenging. Imaging techniques such as ultrasonography, transient elastography, and magnetic resonance imaging provide the reliable detection of liver fat but are not feasible for large-scale screening because of cost, availability, and logistical constraints [11]. Consequently, several non-invasive algorithms based on routinely available clinical and biochemical parameters have been developed to estimate the probability of hepatic steatosis in epidemiological and primary care settings [12].

Among these tools, the Fatty Liver Index (FLI) is one of the most widely used and validated prediction scores. Developed by Bedogni and colleagues, the FLI integrates body mass index (BMI), waist circumference, triglycerides, and gamma-glutamyltransferase levels into a logistic model that estimates the probability of fatty liver [13]. In the original validation study using ultrasonography as the reference standard, FLI values ≥60 were associated with high specificity for hepatic steatosis, whereas values <30 effectively ruled out the condition. Since its introduction, the FLI has been widely applied in large epidemiological cohorts and has demonstrated strong associations with incident diabetes, cardiovascular disease, chronic kidney disease, and mortality [14,15,16].

Central adiposity is a key driver of MASLD pathogenesis. Excess visceral adipose tissue contributes to hepatic lipid accumulation through the increased flux of free fatty acids to the liver, impaired insulin signaling, and activation of inflammatory pathways [17]. Accordingly, anthropometric markers that capture abdominal fat distribution may represent simple and informative tools for identifying individuals at increased risk of MASLD. While BMI remains the most commonly used anthropometric indicator in clinical practice, it does not distinguish between fat and lean mass and provides limited information on body fat distribution [18]. Waist circumference offers a better estimate of abdominal adiposity but may be influenced by body size, sex differences, and ethnic variability [19].

The waist-to-height ratio (WtHR) has been proposed as a potentially superior anthropometric index that standardizes waist circumference relative to stature and therefore better reflects central adiposity across populations. Several epidemiological studies and meta-analyses have suggested that WtHR may outperform BMI and waist circumference in identifying cardiometabolic risk factors, including hypertension, type 2 diabetes, and cardiovascular disease [20,21,22]. The concept that individuals should maintain a waist circumference less than half their height (“keep your waist circumference to less than half your height”) has even been proposed as a simple public health message applicable across ethnicities and age groups [23].

Although increasing evidence supports the role of WtHR as a marker of cardiometabolic risk, its potential utility for identifying individuals with MASLD or MASLD-related phenotypes has not been fully characterized. Previous studies examining the relationship between anthropometric indices and hepatic steatosis have generally been limited by relatively small sample sizes, heterogeneous diagnostic criteria, or lack of detailed subgroup analyses. Furthermore, the optimal WtHR thresholds for identifying individuals at high risk of MASLD remain uncertain.

Large population datasets provide a unique opportunity to better define the relationship between anthropometric markers and MASLD risk. In particular, evaluating WtHR in relation to established surrogate markers of hepatic steatosis may help clarify whether this simple measure could serve as a pragmatic screening tool in clinical and public health contexts.

In this context, the present study aimed to evaluate the performance of waist-to-height ratio as an anthropometric marker for identifying individuals with high Fatty Liver Index, a widely used proxy phenotype of hepatic steatosis, in a large population-based cohort comprising more than 146,000 participants.

Specifically, we sought to (i) assess the discriminatory performance of WtHR using receiver operating characteristic analysis, (ii) determine optimal sex-specific cut-points, (iii) compare its performance with BMI and waist circumference, and (iv) evaluate the robustness of its association with high FLI across different age and BMI strata.

## 2. Materials and Methods

### 2.1. Study Design and Population

This cross-sectional study was conducted using a large population-based dataset comprising 146,318 adult participants with available anthropometric and clinical measurements. The primary aim of the study was to evaluate the performance of waist-to-height ratio (WtHR) as a screening marker for identifying individuals with a high probability of hepatic steatosis as defined by the Fatty Liver Index (FLI).

Participants were recruited as part of routine health assessments conducted in a population-based setting. The dataset included individuals undergoing standardized clinical and anthropometric evaluations, ensuring consistency in measurement procedures. Inclusion criteria required the availability of complete anthropometric and biochemical data necessary to calculate the Fatty Liver Index. Individuals with missing data in any of the variables required for the calculation of the Fatty Liver Index or key anthropometric measures were excluded from the analysis.

Participants were classified according to their FLI category, and individuals with FLI ≥ 60 were categorized as having high FLI, which is commonly interpreted as indicating a high probability of hepatic steatosis in epidemiological studies. The overall prevalence of high FLI in the study population was 18.1%.

### 2.2. Anthropometric Measurements

Anthropometric variables included body mass index (BMI), waist circumference, height, and waist-to-height ratio (WtHR). Body mass index was calculated as weight in kilograms divided by the square of height in meters (kg/m^2^). Waist circumference was measured using standardized anthropometric procedures at the midpoint between the lower margin of the last palpable rib and the iliac crest.

WtHR was calculated as: WtHR = waist circumference/height.

WtHR has been widely proposed as a simple anthropometric indicator reflecting central adiposity and cardiometabolic risk, with several studies suggesting that it may outperform BMI in identifying metabolic abnormalities [24,25].

### 2.3. Fatty Liver Index

The Fatty Liver Index (FLI) was calculated using the original formula proposed by Bedogni et al., which combines BMI, waist circumference, triglycerides, and gamma-glutamyltransferase levels in a logistic regression equation. FLI values range from 0 to 100 and estimate the probability of hepatic steatosis.

According to previously validated thresholds, FLI ≥ 60 indicates a high probability of fatty liver, whereas FLI < 30 rules out hepatic steatosis with high sensitivity [26]. It should be noted that FLI includes anthropometric components (BMI and waist circumference) that are related to WtHR. Therefore, the results should be interpreted considering the potential for mathematical coupling between the predictor and outcome.

In addition, FLI is a surrogate marker of hepatic steatosis and does not replace imaging-based or histological diagnosis. This should be considered when interpreting the results.

### 2.4. Statistical Analysis

Continuous variables were summarized as the median and interquartile range (IQR) due to non-normal distribution, whereas categorical variables were presented as counts and percentages.

### 2.5. Discrimination Analysis

The discriminatory ability of WtHR to identify individuals with high FLI was assessed using receiver operating characteristic (ROC) curve analysis. The area under the ROC curve (AUC) was calculated with 95% confidence intervals using the DeLong method, which provides a nonparametric estimate of the variance of correlated ROC curves [27].

Optimal WtHR thresholds were identified using the Youden index, defined as the value maximizing sensitivity + specificity − 1 [28].

### 2.6. Comparative Performance Analysis

To compare the performance of different anthropometric indicators, ROC curves were also generated for BMI and waist circumference. Pairwise comparisons between AUCs were performed using the paired DeLong test for correlated ROC curves [27].

### 2.7. Logistic Regression Analysis

Sex-stratified logistic regression models were used to evaluate the association between WtHR and the probability of high FLI. Models were adjusted for age. Additional metabolic variables were not included in the primary models, as the aim of the study was to evaluate the discriminatory performance of WtHR as a simple anthropometric screening tool. Including such variables could reduce the practical applicability of the model in population-level screening settings. Results were expressed as odds ratios (ORs) per 0.01 increase in WtHR with corresponding 95% confidence intervals.

Logistic regression models are widely used in epidemiological studies to estimate associations between risk factors and binary outcomes [29].

### 2.8. Model Calibration

Model calibration was assessed by comparing the predicted and observed probabilities of high FLI across deciles of predicted risk.

### 2.9. Restricted Cubic Spline Analysis

To explore potential non-linear relationships between WtHR and the probability of high FLI, restricted cubic spline models were fitted within logistic regression frameworks. Knots were placed at the 5th, 35th, 65th, and 95th percentiles of the WtHR distribution, a commonly recommended approach for modeling non-linear associations in epidemiological data [30].

Non-linearity was evaluated using likelihood ratio tests comparing spline models with linear models.

### 2.10. Risk Category Analysis

WtHR values were categorized into four clinically interpretable groups:

- <0.45;
- 0.45–0.50;
- 0.50–0.55;
- ≥0.55.

The prevalence of high FLI was calculated within each category, and logistic regression models were used to estimate age-adjusted odds ratios.

#### Subgroup Analysis

Subgroup analyses were conducted to evaluate the robustness of the association between WtHR and high FLI across different demographic and anthropometric strata. Analyses were stratified by:

- Sex;
- Age groups (<40, 40–49, 50–59, ≥60 years);
- BMI categories.

Interaction between WtHR and sex was formally tested using interaction terms in logistic regression models.

### 2.11. Decision Curve Analysis

Decision curve analysis (DCA) was performed to evaluate the potential clinical utility of WtHR for identifying individuals with high FLI. Net benefit was calculated across a range of threshold probabilities and compared with models including BMI and waist circumference. Decision curve analysis is increasingly used to assess the clinical value of predictive models beyond traditional discrimination metrics [31].

### 2.12. Statistical Software

All statistical analyses were conducted using SPSS version 30.0 (IBM Corp., Armonk, NY, USA) and R statistical software. A two-sided *p*-value < 0.05 was considered statistically significant.

Analyses were conducted using a complete-case approach, including only participants with available data for all variables required in the study. The proportion of missing data was low, and no imputation procedures were applied. Therefore, individuals with missing values in key variables were excluded from the analysis.

No formal sensitivity analyses were performed.

## 3. Results

### 3.1. Population Characteristics

The study included a total of 146,318 individuals, of whom 26,546 (18.1%) were classified as having high Fatty Liver Index (FLI ≥ 60). Men represented 83,602 participants, with a prevalence of high FLI of 25.3%, whereas women accounted for 62,716 participants, with a prevalence of 8.6%.

Baseline characteristics of the study population according to FLI category are presented in Table 1.

Individuals with high FLI were older and had markedly higher waist-to-height ratio (WtHR) values compared with those without high FLI. Median WtHR values were 0.470 [0.430–0.500] in individuals without high FLI and 0.560 [0.540–0.590] in those with high FLI.

Similarly, FLI values differed substantially between groups, with median values of 15.61 [6.79–31.75] among individuals without high FLI and 77.30 [68.01–87.36] among those with high FLI.

#### Discriminatory Performance of WtHR

The discriminatory performance of WtHR for identifying individuals with high FLI was evaluated using receiver operating characteristic (ROC) curve analysis stratified by sex.

WtHR demonstrated excellent discrimination, with an area under the curve (AUC) of:

- 0.908 (95% CI 0.906–0.910) in men;
- 0.972 (95% CI 0.971–0.974) in women.

**Figure 1:**
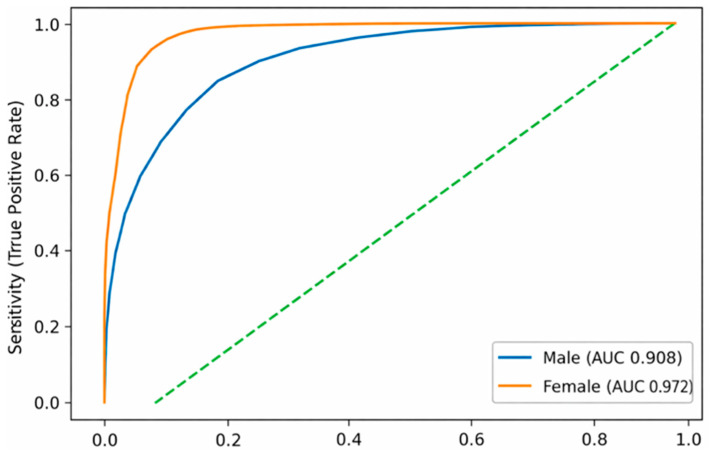
ROC curves of waist-to-height ratio for identifying high FLI by sex.

These findings indicate a very high ability of WtHR to identify individuals with a high-FLI phenotype, particularly among women. (Figure 1).

To evaluate the discriminatory ability of waist-to-height ratio (WtHR) for identifying individuals with high Fatty Liver Index (FLI), receiver operating characteristic (ROC) curves were constructed separately for men and women.

The figure displays ROC curves showing the relationship between sensitivity and 1–specificity across the full range of WtHR values. The area under the curve (AUC) was 0.908 (95% CI 0.906–0.910) in men and 0.972 (95% CI 0.971–0.974) in women, indicating excellent discrimination. These results demonstrate that WtHR can accurately identify individuals with a high-FLI phenotype, particularly among women.

### 3.2. Optimal WtHR Cut-Points

Optimal WtHR thresholds were determined using the Youden index, which identifies the value maximizing the combined sensitivity and specificity.

The optimal WtHR cut-points for identifying individuals with high FLI were 0.520 in men and 0.530 in women. The diagnostic performance of these thresholds is summarized in Table 2.

These thresholds provided high diagnostic accuracy for identifying individuals with high FLI.

### 3.3. Association Between WtHR and High FLI

Sex-stratified logistic regression models adjusted for age demonstrated a strong positive association between WtHR and the probability of high FLI.

Each 0.01 increase in WtHR was associated with:

- OR 1.56 (95% CI 1.55–1.57) in men;
- OR 1.69 (95% CI 1.67–1.72) in women.

These results indicate that relatively small increases in WtHR are associated with substantial increases in the likelihood of presenting a high-FLI phenotype.

### 3.4. Comparison with BMI and Waist Circumference

The discriminatory performance of WtHR was compared with BMI and waist circumference using paired ROC analysis.

In the overall cohort, AUC values were:

- WtHR: 0.933 (95% CI 0.931–0.934);
- BMI: 0.932 (95% CI 0.930–0.933);
- Waist circumference: 0.932 (95% CI 0.931–0.934).

The discriminatory performance of WtHR was compared with the BMI and waist circumference using paired ROC analysis. In the overall cohort, the AUC values are summarized in Table 3.

Pairwise comparisons using the DeLong test showed no statistically significant differences between WtHR and the other anthropometric indicators in the overall population.

Sex-specific analyses revealed some differences. In men, BMI showed slightly higher discrimination than WtHR, whereas WtHR performed better than waist circumference. In women, BMI showed the highest AUC, while WtHR demonstrated similar discrimination to waist circumference.

### 3.5. Evaluation Through Decision Curve Analysis

Decision curve analysis was performed to evaluate the clinical utility of WtHR for identifying individuals with high FLI.

Across a broad range of threshold probabilities, the WtHR-based model showed net benefit comparable to models based on BMI and waist circumference, suggesting that WtHR may provide similar clinical utility as a screening indicator for identifying individuals at increased risk of MASLD.

#### Model Calibration

**Figure 2:**
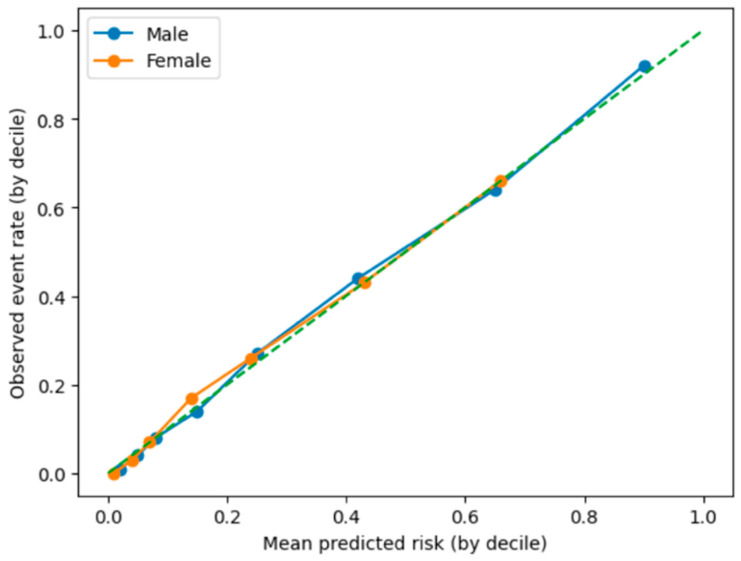
Calibration plot of the WtHR + age model for predicting high FLI. Model calibration was assessed to evaluate the agreement between the predicted and observed probabilities of high FLI.

Model calibration analysis showed good agreement between the predicted and observed probabilities of high FLI across deciles of predicted risk (Figure 2).

The calibration plot compares the predicted risk with observed prevalence of high FLI across deciles of predicted probability. Points close to the diagonal line indicate good calibration, meaning that the model provides reliable estimates of the true probability of presenting a high-FLI phenotype.

### 3.6. Non-Linear Association Between WtHR and High FLI

Restricted cubic spline models were used to explore the relationship between WtHR and the probability of high FLI.

Restricted cubic spline analysis demonstrated a non-linear association between WtHR and the predicted probability of high FLI in men (likelihood ratio χ^2^ = 86.5, df = 4, *p* < 0.001).

**Figure 3:**
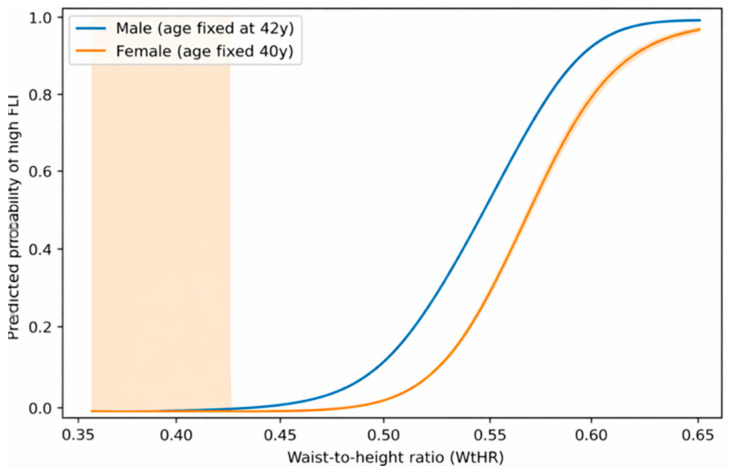
Age-adjusted predicted probability of high FLI according to waist-to-height ratio.

The predicted probability of high FLI increased sharply once WtHR values exceeded approximately 0.50–0.52, suggesting a threshold effect in the relationship between abdominal adiposity and MASLD risk (Figure 3).

To explore the shape of the association between WtHR and the probability of high FLI, logistic regression models with restricted cubic splines adjusted for age were fitted.

Figure 3 shows the predicted probability of high FLI across the distribution of WtHR values. The curve demonstrated a clear non-linear association, with a steep increase in predicted probability once WtHR exceeded approximately 0.50–0.52. This pattern suggests the presence of a threshold effect linking central adiposity with increased metabolic liver risk.

#### 3.6.1. Risk Categories of WtHR

WtHR values were categorized into four clinically interpretable groups:

- <0.45;
- 0.45–0.50;
- 0.50–0.55;
- ≥0.55.

WtHR values were categorized into four clinically interpretable groups (<0.45, 0.45–0.50, 0.50–0.55, and ≥0.55). A strong gradient in the prevalence of high FLI was observed across these categories (Table 4).

Among men, the prevalence of high FLI increased from:

- 0.7% for WtHR < 0.45 to 82.5% for WtHR ≥ 0.55.

Among women, the prevalence increased from:

- 0.0% for WtHR < 0.45 to 66.8% for WtHR ≥ 0.55.

Age-adjusted logistic regression analyses showed progressively higher odds of high FLI with increasing WtHR categories. Compared with WtHR < 0.45, the odds ratios were:

- OR 15.21 (95% CI 12.81–18.06) for WtHR 0.45–0.50;
- OR 138.74 (95% CI 117.31–164.09) for WtHR 0.50–0.55;
- OR 1219.21 (95% CI 1029.26–1444.20) for WtHR ≥ 0.55.

#### 3.6.2. Subgroup Analyses

Subgroup analyses were conducted to evaluate the robustness of the association between WtHR and high FLI across age and BMI strata.

Across age groups, WtHR retained strong discriminatory performance (Table 5). In men, the AUC ranged from 0.872 to 0.929, whereas in women, it ranged from 0.954 to 0.980, indicating consistently high discrimination across different age categories.

Similarly, positive associations between WtHR and high FLI were observed across BMI categories (Table 6). Discrimination was lower among individuals with normal BMI compared with those with overweight or obesity.

Formal interaction testing demonstrated significant effect modification by sex (*p* = 8.4 × 10^−32^).

## 4. Discussion

In this large population-based study including more than 146,000 individuals, waist-to-height ratio (WtHR) showed strong discriminatory ability for identifying individuals with a high Fatty Liver Index (FLI), a widely used surrogate marker of hepatic steatosis. These findings reinforce the growing evidence that central adiposity plays a central role in the pathophysiology of metabolic dysfunction-associated steatotic liver disease (MASLD) and that anthropometric indicators capturing abdominal fat distribution may be particularly useful for identifying individuals at increased metabolic liver risk [32].

The very high AUC values observed in this study, particularly among women, should be interpreted with caution. This finding may partly reflect the use of FLI as the outcome, which shares anthropometric components with the predictor, potentially contributing to an overestimation of discriminatory performance. In addition, the relatively homogeneous characteristics of the study population and the strong relationship between central adiposity and FLI may have further enhanced model performance. These factors should be considered when interpreting the magnitude of the observed AUC values.

### 4.1. Key Findings

The present study provides several important findings. First, waist-to-height ratio demonstrated excellent discriminatory performance for identifying individuals with a high Fatty Liver Index in a very large population-based cohort. Second, we identified sex-specific WtHR thresholds of approximately 0.52 in men and 0.53 in women for detecting individuals with high FLI. Third, restricted cubic spline analyses revealed a non-linear association between WtHR and the probability of high FLI, with a sharp increase in risk above values of approximately 0.50–0.52. Finally, WtHR demonstrated discriminatory performance comparable to BMI and waist circumference, while maintaining strong associations with elevated FLI across different age groups and BMI categories, with no statistically significant differences. These findings suggest that its primary advantage lies in its simplicity and ease of use rather than in superior predictive performance. Nevertheless, these thresholds are consistent with prior literature indicating that WtHR values around 0.50 are associated with increased cardiometabolic risk.

The close relationship between visceral adiposity and hepatic steatosis has been consistently demonstrated in both experimental and epidemiological studies. Excess visceral fat promotes hepatic lipid accumulation through the increased delivery of free fatty acids to the liver, impaired insulin signaling, and activation of inflammatory pathways that contribute to metabolic dysfunction and steatosis progression [33,34,35]. In this context, anthropometric indicators reflecting central adiposity may capture the pathophysiological mechanisms directly involved in MASLD development.

Our results are consistent with previous studies showing that indices of abdominal adiposity are strongly associated with fatty liver disease. Large population studies have reported that anthropometric measures incorporating waist circumference are closely linked with hepatic steatosis and metabolic risk. For example, analyses from the UK Biobank have demonstrated that central obesity markers are strongly associated with fatty liver disease and cardiometabolic complications, even after accounting for overall adiposity [36]. Similarly, recent epidemiological studies have shown that waist-based indices may outperform BMI in identifying individuals with metabolic liver disease and related metabolic abnormalities [37].

One of the key findings of our study is the identification of sex-specific WtHR thresholds of approximately 0.52 in men and 0.53 in women as optimal cut-points for identifying individuals with high FLI. These values are broadly consistent with previous research suggesting that WtHR values around 0.50 represent a clinically meaningful boundary beyond which the cardiometabolic risk increases substantially [38]. However, our findings suggest that slightly higher thresholds may better identify individuals with metabolic liver disease risk. This observation is consistent with previous studies indicating that the relationship between abdominal adiposity and metabolic complications may vary according to sex and population characteristics [39,40].

Another notable observation is the strong dose–response relationship between WtHR and the probability of high FLI. Individuals in the highest WtHR categories exhibited a markedly higher prevalence of high FLI compared with those with lower ratios. This gradient supports the concept that increasing central adiposity is closely linked with worsening metabolic dysfunction and hepatic fat accumulation. Similar patterns have been described in recent population studies evaluating the association between abdominal obesity and MASLD prevalence [41].

The non-linear association observed in the spline analyses further supports the presence of a threshold effect linking abdominal adiposity with hepatic steatosis risk. In our cohort, the probability of high FLI increased sharply once WtHR exceeded approximately 0.50–0.52. This pattern is biologically plausible and may reflect the point at which adipose tissue storage capacity becomes insufficient, leading to ectopic lipid deposition in organs such as the liver. Experimental studies have shown that once adipose tissue expandability is exceeded, excess lipids accumulate in non-adipose tissues, contributing to metabolic dysfunction and hepatic steatosis [42].

Importantly, the association between WtHR and high FLI remained consistent across age groups and BMI categories. Even among individuals with normal BMI, increasing WtHR was associated with higher probability of high FLI. This observation highlights the limitation of BMI as a marker of metabolic risk and supports the concept that individuals with normal body weight but increased visceral adiposity may still develop metabolic complications. The phenomenon of “lean MASLD” has been increasingly recognized and is thought to reflect unfavorable fat distribution rather than overall adiposity alone [43].

From a clinical and public health perspective, WtHR represents an attractive screening indicator because of its simplicity and accessibility. Unlike composite biochemical scores used to estimate liver fat, WtHR can be calculated using only waist circumference and height and does not require laboratory measurements. In settings where imaging-based screening is not feasible, simple anthropometric markers may therefore help identify individuals who could benefit from further metabolic evaluation or targeted lifestyle interventions [44].

### 4.2. Public Health Implications

From a public health perspective, these findings are particularly relevant given the rapidly increasing global burden of MASLD. Screening strategies based on imaging or laboratory biomarkers are difficult to implement at the population level, particularly in primary care or resource-limited settings. Simple anthropometric indicators such as waist-to-height ratio may therefore offer a pragmatic alternative for the early identification of individuals at increased metabolic liver risk. Because WtHR requires only waist circumference and height measurements, it can be easily incorporated into routine clinical assessments and population-based health programs aimed at preventing metabolic diseases.

### 4.3. Limitations

Nevertheless, several limitations should be considered when interpreting our findings. A key methodological limitation of this study relates to the definition of the outcome variable. The Fatty Liver Index (FLI), used to identify individuals with a high probability of hepatic steatosis, incorporates body mass index and waist circumference among its components. Because these variables are intrinsically related to the main predictor (waist-to-height ratio), this introduces a risk of mathematical coupling and potential circular reasoning. As a result, the observed discriminatory performance (AUC) of WtHR may be partially inflated due to the shared structure between the predictor and outcome.

Therefore, our findings should be interpreted as reflecting the ability of WtHR to identify a high-FLI phenotype rather than providing an independent validation of hepatic steatosis. Although FLI is a widely used and validated surrogate marker in epidemiological studies, it does not replace imaging-based diagnosis. Future studies should prioritize the validation of WtHR against independent reference standards, such as imaging-confirmed steatosis using transient elastography (FibroScan), ultrasound, or magnetic resonance imaging, in order to better establish its clinical utility. This limitation has been highlighted in previous methodological discussions of fatty liver indices and should be considered when interpreting discrimination metrics [45].

Third, the cross-sectional nature of the study prevents conclusions regarding causal relationships or the longitudinal prediction of MASLD. Prospective studies will be required to determine whether WtHR can predict incident MASLD or progression of liver disease over time.

Another limitation of this study is the lack of external validation. Although the analysis was conducted in a very large population-based cohort, all results were derived from a single population. Therefore, the proposed WtHR cutoffs may not be directly generalizable to other populations with different ethnic, clinical, or metabolic characteristics. External validation in independent cohorts is required to confirm the robustness and applicability of these thresholds across diverse settings. In addition, variations in body fat distribution, metabolic risk, and anthropometric performance across different ethnic groups and healthcare settings may further limit the generalizability of these findings.

Another limitation is that regression models were adjusted only for age and did not include additional potential confounders such as metabolic comorbidities, medication use, or biochemical parameters. While this approach was intentional to preserve the simplicity and applicability of WtHR as a screening tool, residual confounding cannot be excluded.

In addition, the use of complete-case analysis may introduce selection bias if excluded individuals differ systematically from those included in the analysis. This should be considered when interpreting the findings.

Furthermore, no sensitivity analyses were conducted to assess the robustness of the findings under different model specifications, which may limit the evaluation of the stability of the results.

Despite these limitations, the present study provides robust evidence supporting the use of waist-to-height ratio as a simple anthropometric marker associated with a high-FLI phenotype in a very large population-based cohort. Given its simplicity, low cost, and strong association with metabolic liver risk, WtHR may represent a practical tool for identifying individuals at increased risk of MASLD in population and primary care settings.

## 5. Conclusions

In this large population-based study including more than 146,000 individuals, waist-to-height ratio (WtHR) demonstrated strong discriminatory ability for identifying individuals with a high Fatty Liver Index (FLI), a widely used surrogate marker of hepatic steatosis. WtHR showed excellent performance in both men and women and maintained robust associations with high FLI across different age groups and BMI categories.

Optimal sex-specific thresholds of approximately 0.52 in men and 0.53 in women were identified for detecting individuals with high FLI, suggesting that relatively small increases in WtHR are associated with a substantially higher probability of presenting a high-FLI phenotype. The non-linear association observed in spline analyses further supports the presence of a threshold effect linking central adiposity with increased metabolic liver risk.

Importantly, WtHR showed discriminatory performance comparable to BMI and waist circumference while offering a simple anthropometric indicator that can be calculated without laboratory measurements. Given its simplicity, low cost, and ease of implementation, WtHR may represent a practical screening tool for identifying individuals with a high-FLI phenotype in both clinical practice and large-scale population health strategies.

Taken together, these findings support the potential role of waist-to-height ratio as a pragmatic anthropometric marker for population-level risk stratification of MASLD-related phenotypes. Considering the growing global burden of MASLD, simple anthropometric indicators such as WtHR may help facilitate the early identification of individuals at increased metabolic liver risk. Future prospective studies are warranted to determine whether WtHR can predict incident MASLD and long-term liver-related outcomes.

These findings should be interpreted in light of the use of a surrogate outcome and require confirmation using imaging-based measures of hepatic steatosis.

## Figures and Tables

**Table 1:** Baseline characteristics of the study population according to Fatty Liver Index category.

Variable	Not High FLI (*n* = 119,772)	High FLI (*n* = 26,546)
Age (years)	40 [32–48]	44 [37–51]
WtHR	0.470 [0.430–0.500]	0.560 [0.540–0.590]
FLI	15.61 [6.79–31.75]	77.30 [68.01–87.36]

Data are presented as median [interquartile range]. FLI = Fatty Liver Index; WtHR = waist-to-height ratio.

**Table 2:** Diagnostic performance of optimal WtHR cut-points for identifying high FLI.

Sex	WtHR Cut-Point	Sensitivity (%)	Specificity (%)	PPV (%)	NPV (%)
Men	0.520	85.8	80.2	60.5	94.1
Women	0.530	93.9	90.2	47.6	99.3

Optimal thresholds were determined using the Youden index from ROC curve analysis. WtHR = waist-to-height ratio; FLI = Fatty Liver Index. PPV = positive predictive value. NPV = negative predictive value.

**Table 3:** Discriminatory performance of anthropometric indicators for identifying individuals with high FLI.

Predictor	*n*	Events	AUC	95% CI	*p*-Value vs. WtHR
BMI	146,318	26,546	0.932	0.930–0.933	0.18
Waist circumference	146,318	26,546	0.932	0.931–0.934	0.27
WtHR	146,318	26,546	0.933	0.931–0.934	Reference

AUC values were calculated using the DeLong method. AUC = area under the ROC curve. BMI = body mass index. WtHR = waist-to-height ratio.

**Table 4:** Prevalence and odds ratios for high FLI according to WtHR categories stratified by sex.

WtHR Category	Men: High FLI (%)	Women: High FLI (%)	OR (95% CI)
<0.45	0.7	0.0	Reference
0.45–0.50	6.4	0.9	15.21 (12.81–18.06)
0.50–0.55	34.3	16.1	138.74 (117.31–164.09)
≥0.55	82.5	66.8	1219.21 (1029.26–1444.20)

Odds ratios were obtained from age-adjusted logistic regression models. WtHR = waist-to-height ratio; FLI = Fatty liver index. OR = Odds ratio. CI = Confidence interval.

**Table 5:** Discriminatory performance of WtHR for identifying high FLI across age subgroups stratified by sex.

Subgroup	*n*	Events (High FLI)	AUC	95% CI
Men < 40 years	34,278	6330	0.929	0.926–0.932
Men 40–49 years	28,128	8154	0.901	0.897–0.905
Men 50–59 years	17,970	5672	0.896	0.891–0.901
Men ≥ 60 years	3226	1012	0.872	0.859–0.885
Women < 40 years	30,306	2180	0.980	0.979–0.982
Women 40–49 years	20,082	1738	0.973	0.970–0.975
Women 50–59 years	10,516	1242	0.954	0.949–0.959
Women ≥ 60 years	1812	218	0.962	0.952–0.972

AUC values were calculated using ROC curve analysis with 95% confidence intervals computed by the DeLong method. AUC, area under the curve; FLI, Fatty Liver Index; WtHR, waist-to-height ratio.

**Table 6:** Discriminatory performance of WtHR for identifying high FLI across BMI categories.

BMI Category	*n*	High FLI (*n*)	AUC	95% CI
Normal weight (<25 kg/m^2^)	62,148	2318	0.861	0.854–0.868
Overweight (25–29.9 kg/m^2^)	58,304	13,402	0.914	0.910–0.918
Obesity (≥30 kg/m^2^)	25,866	10,826	0.902	0.897–0.907

AUC values were calculated using ROC curve analysis with 95% confidence intervals estimated by the DeLong method. BMI = body mass index. FLI = Fatty Liver Index. WtHR = waist-to-height ratio. AUC = area under the curve.

## Data Availability

The datasets generated and analyzed during the present study are securely maintained at ADEMA University School. Data management and storage are carried out in accordance with current data protection regulations and are supervised by the institution’s Data Protection Officer, Ángel Arturo López González.

## Abbreviations

The following abbreviations are used in this manuscript:

AUC	Area Under the Curve
BMI	Body Mass Index
CEI-IB	Research Ethics Committee of the Balearic Islands
CI	Confidence Interval
DCA	Decision Curve Analysis
FLI	Fatty Liver Index
IQR	Interquartile Range
MASLD	Metabolic Dysfunction-Associated Steatotic Liver Disease
NAFLD	Non-Alcoholic Fatty Liver Disease
NPV	Negative Predictive Value
OR	Odds Ratio
PPV	Positive Predictive Value
ROC	Receiver Operating Characteristic
WtHR	Waist-to-Height Ratio
